# Chagas Disease Diagnostic Testing in Two Academic Hospitals in New Orleans, Louisiana: A Call to Action

**DOI:** 10.3390/tropicalmed8050277

**Published:** 2023-05-15

**Authors:** Alvaro Proaño, Eric Dumonteil, Claudia Herrera

**Affiliations:** 1 Department of Pediatrics, Tulane University School of Medicine, New Orleans, LA 70112, USA; aproano@tulane.edu; 2 Department of Tropical Medicine, Vector-Borne and Infectious Disease Research Center, School of Public Health and Tropical Medicine, Tulane University, New Orleans, LA 70112, USA; edumonte@tulane.edu

**Keywords:** *Trypanosoma cruzi*, diagnostic, surveillance, screening, health access, chagas disease barriers

## Abstract

Chagas disease, caused by the protozoa parasite *Trypanosoma cruzi*, is an anthropozoonosis that represents a major public health problem in the Americas, affecting 7 million people with at least 65 million at risk. We sought to assess the intensity of disease surveillance based on diagnostic test requests from hospitals in New Orleans, Louisiana. We extracted information from send-out labs at two major tertiary academic hospitals in New Orleans, Louisiana, USA, from 1 January 2018 to 1 December 2020. We found that in these three years there were 27 patients for whom Chagas disease testing was ordered. Most of these patients were male (70%), with a median age of 40 years old, and their most common ethnical background was Hispanic (74%). These findings demonstrate undertesting of this neglected disease in our region. Given the low Chagas disease surveillance, we need to increase awareness, health promotion, and education among healthcare workers.

## 1. Introduction

Chagas disease is a neglected tropical disease and a serious public health issue in Latin America and increasingly in the United States of America (US). In the US, the burden of Chagas disease incurs a cost of USD 0.9 billion dollars each year [1]. Chagas disease results from infection with the protozoan parasite *Trypanosoma cruzi*, affecting 6–8 million people with at least 65–100 million people at risk [2]. Transmission in endemic countries, including in the US, is mainly vectorial, although vertical transmission and blood donations are taking a more relevant role. In the US, there is a rough estimate of 300,000 people living with Chagas disease, making it the sixth country in disease burden worldwide [3,4,5,6]. Despite this disease burden, in the US, less than 1% of domestic cases have been identified and less than 0.3% received treatment [5].

There is a lack of systematic screening of the disease, as well as a lack of awareness by medical providers; thus, it remains a neglected disease [5,7,8]. The exact number of patients living with this disease in the US is unknown given that there is no national surveillance and the latest national estimate from 2012 did not take into account undocumented immigrants, which account for at least 10.5 million people in the country [9,10]. The disease is thus likely underestimated, largely due a lack of knowledge from health providers and the general population [11]. Regarding obstetric care, Chagas disease remains an important disease given that it can be transmitted vertically to newborns by their mother [12]. In the US, there is an estimated 65–315 congenital cases of Chagas disease per year, which is within the range of other important congenital diseases such as phenylketonuria, with 254 births per year, and congenital adrenal hyperplasia, with 121 births per year [3]. Maternal–fetal transmission may be repeated in each pregnancy for that same family and can also be transmitted from mother to child, passing from one generation to another [13]. If left untreated, it can have devastating consequences to multiple organ systems including cardiac, digestive, and neurologic [14,15,16].

The disease is still underestimated, due in part to a lack of knowledge from health providers and the general population [11]. New cases are being identified in the US, and this is most likely due to screening being performed in blood donor centers; however, blood donor screening stopped in February 2020 [17,18]. Reporting of Chagas disease has been mandatory only in seven states, including Arizona, Arkansas, Louisiana, Massachusetts, Mississippi, Tennessee, and Texas, although cases have been estimated in every state [9,19]. In Louisiana, the Department of Health deems this disease as endemic, and its first human case was reported in 2006 [20,21].

Autochthonous human cases of Chagas disease have been reported in the US [22]. In Louisiana, a total of at least 15 new cases have been reported based on information from the Louisiana Department of Health and the Association for the Advancement of Blood & Biotherapies (AABB, formerly known as the American Association of Blood Banks) Chagas Biovigilance Network [18,21]. Additionally, it has recently been shown that *Trypanosoma cruzi* is prevalent in wildlife within Louisiana, including rodents and racoons that would act as reservoirs for this pathogen [23,24]. There is also a report that evaluated the vector *Triatoma sanguisuga* in Louisiana and found that at least 55% of them were infected with *Trypanosoma cruzi* and that nearly 40% of them fed on humans [25]. Finally, there were also cases from a hospital in New Orleans that showed chronic Chagas disease presenting itself with cardiac manifestations in two patients [26]. All this signals that there is Chagas disease in New Orleans; however, no prior study has evaluated the frequency of tests being ordered regarding Chagas disease in the New Orleans region. We aimed to assess the intensity of disease surveillance based on diagnostic test requests from major hospitals in New Orleans, Louisiana.

## 2. Materials and Methods

### 2.1. Study Design

We retrospectively evaluated electronic medical records of a three-year period (2018–2020), similar to a recent study [27].

### 2.2. Setting

We extracted information from send-out labs at the University Medical Center in New Orleans (UMCNO) and from the Children’s Hospital New Orleans (CHNOLA) from 1 January 2018 to 1 December 2020. UMCNO is a 446-bed adult academic hospital, and CHNOLA is a 257-bed pediatric academic hospital, both in the New Orleans metropolitan area.

Both hospitals provide tertiary-level care for the New Orleans region in Louisiana for adult and pediatric care, respectively. In 2020, UMCNO provided 154,372 clinic visits and 74,143 emergency room visits. In 2020, CHNOLA provided 183,471 clinic visits and 33,670 emergency room visits.

### 2.3. Data Extraction

We partnered with the send-out labs at UMCNO and CHNOLA. They retrieved information from their laboratory system. They evaluated all the tests that have been ordered that contained the words “trypanosoma”, “cruzi”, or “chagas”.

They retrieved information regarding each patient’s age at the time of testing, the biological sex, the patient’s ethnicity, the type of the Chagas test that was ordered, and the test result.

### 2.4. Ethics

This study received an IRB exemption from Tulane University (IRB # 2020-2207) after determining it was not human subject research.

## 3. Results

We searched for ordered tests related to Chagas disease during this time period by free-text search of orders through the send-out lab. In these three years, there were a total of 39 Chagas disease tests from 27 patients that were ordered, and most tests were ordered at UMCNO (84%), as shown in Table 1. There were two additional tests that were ordered and cancelled that were not included in this report. Most orders were sent to ARUP Laboratories (88%) and the rest to Centers for Disease Control and Prevention (CDC).

**Figure 1:**
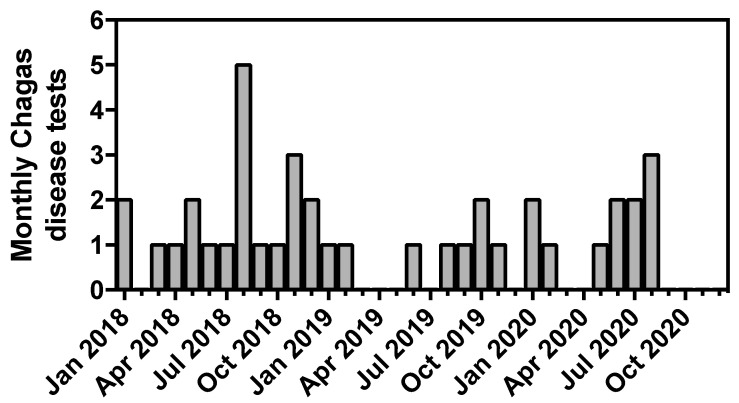
Number of Tests Requested Monthly from January 2018 to December 2020.

Most requests were for immunoglobulin G (IgG) or immunoglobulin M (IgM), and only one was for polymerase chain reaction (PCR). Usually, one or two tests were sporadically ordered per month throughout the study period (Figure 1). An average of nine patients per year were tested. Patients’ demographics are shown in Table 1, and one patient declined his ethnicity being reported. Most were male (70%), the age range was from 4 to 75 years old (median 40 years), and their most common ethnical background was Hispanic (74%). Seven patients had multiple tests performed, and 71% of these patients were Hispanic and 71% were male.

Two patients resulted positive initially, both at UMCNO, which gives a presumed seroprevalence rate of 7.4% (95% confidence interval [2.3–23.5%]). They were both Hispanic, one a 41-year-old male and the other a 62-year-old female. The first patient (41-year-old male) had an initial IgM test that was positive, but follow-up tests were then negative for both IgG and IgM both in ARUP and CDC. It is unknown if this patient was treated in between tests. The second patient (62-year-old female) was IgG-positive by ARUP, but no confirmatory test was performed. No pediatric cases were found to be positive.

## 4. Discussion

Our results highlight a strong undertesting for Chagas disease at two major New Orleans Hospitals despite a likely high prevalence in our region. Two similar studies evaluating the disease frequency have been performed, one in San Francisco, California, between 2016 and 2019 and the other in Denver, Colorado, between 2006 and 2020. At the University of San Francisco Hospital in California, they found that 109 patients were evaluated for Chagas disease in a 3-year span, accounting for 36 patients per year [27]. At the University of Colorado Hospital, 1156 patients were evaluated for Chagas disease in a 14-year span, which is 83 patients per year [28]. New Orleans had the least number of tests ordered (9 patients per year in New Orleans vs. 36 patients per year in San Francisco vs. 83 patients per year in Denver). Seroprevalence across these studies is not directly comparable given that not all patients who were screened positive in each site had confirmatory results, but the data suggest a high prevalence in New Orleans (7% in New Orleans vs. 3% in San Francisco vs. 0.03% in Denver). To our knowledge, a national study evaluating diagnostic frequency of Chagas disease has not been conducted. We were not able to evaluate country of origin, but the San Francisco and the Denver studies showed that their confirmed positive cases came exclusively from those born in Latin America [27]. In New Orleans, the number of Hispanics has been increasing in the past decades, from 58,545 (4% of the population) in 2000 to 116,254 (9% of the population) in 2020 [29]. Therefore, with the increasing number of Hispanics in the region, we expect Chagas disease to become more prevalent; thus, its testing is becoming more urgent in this region. A study in 2015 estimated the number of people living with Chagas disease in each state and estimated that there were 1,427 cases in Louisiana and reported that the AABB Chagas Biovigilance Network found 15 cases in this state [9]. However, blood donor screening surveillance stopped in 2020, and it has been considered that it underestimates the seroprevalence of the disease due to the low number of foreign-born Hispanics who donate blood [19]. Additionally, a recently published map (in 2022) estimated the disease per region in the US and found that in the Orleans parish about 2.06% of Latin-American born adults live with Chagas disease [30].

Regarding congenital infections, our results did not find any, and no tests were ordered in the neonatal period; however, only two congenital cases have been reported in the US out of an estimated of 60–315 per year [3,6]. One was reported in Washington, DC, and the other one in Virginia [31,32]. Recently, in Florida, two siblings were diagnosed with Chagas disease during their adulthood (24 and 26 year old) who are presumed to have been infected congenitally given that their mother was also positive and there were no other potential sources of infection [33]. No cases have been reported in Louisiana, but this could potentially be due to lack of testing.

A limitation of our study includes that this was a de-identified study that did not evaluate which service ordered the Chagas tests, and there was no chart review involved. Thus, we could not identify the reasons why there were patients that had multiple tests ordered. Further studies should be conducted to determine which providers are ordering these tests and evaluate if there is a correlation regarding awareness of the disease and test ordering. We also did not have data of where Hispanics are from in our study; however, estimates from the Orleans parish show that most are from Honduras or Mexico [29]. Another limitation of our study is that it did not include a labor and delivery hospital; thus, evaluating congenital Chagas disease was limited. However, we included two of the largest tertiary care hospitals in the New Orleans region, UMCNO and CHNOLA, serving the pediatric and the adult population. Another limitation is that we do not have information determining if one of our initially positive patients was a false-positive; we also do not have a confirmatory test for our second patient who was positive.

Forsyth and collaborators explored multiple barriers that could be causing this abysmal difference between estimated people living with Chagas disease in the US and those being identified [5]. These barriers include structural, systemic, clinical, and psychosocial barriers. Structural barriers include multiple intrinsic problems Hispanics face including coming from endemic countries, poverty, and lack of healthcare access. Systemic barriers include lack of awareness of the disease by physicians, limited testing options, and limited access to medications. Multiple studies in the US have shown the lack of awareness of this disease by different groups of physicians, including obstetrician–gynecologists and pediatric infectious disease specialists [34,35]. Clinical barriers include difficulties with diagnosis including lack of tests developed specifically for the US population, difficulties with treatment monitoring, and lack of knowledge regarding tolerability of medications. A recent study by Mahoney West et al. showed that, despite the fact that most providers considered Chagas disease to be of importance, only one third of them knew how to order a test and less than a third knew what to do next if the test was positive [7]. Finally, psychosocial barriers include stigmatization of an already vulnerable population in the US: immigrants.

We also need to specifically address the lack of congenital cases that have been reported in the US and in our study. This is important given that estimates show that 43,000 women of reproductive age in the US live with Chagas disease and given that the parasite can be transmitted vertically in each pregnancy [12,13,30]. To our knowledge, there are three studies that have evaluated the maternal prevalence of Chagas disease in the US. One was conducted in Houston, Texas, between 1993 and 1999 and found a prevalence of 0.3% (11/3765), and from 2011 to 2012 a similar study found a prevalence of 0.25% (10/4000) [36,37]. In Boston, Massachusetts, between 2018 and 2019 a targeted screening reported a prevalence of 0.5% (3/619) [38] In the US, it is estimated that maternal/infant screening would provide USD 634 million societal savings per birth cohort [39]. A recent guideline by Chagas experts in the US recommends screening women of childbearing age [40]. However, a survey by the American College of Obstetricians and Gynecologists demonstrated that most members never considered Chagas disease as a diagnosis from their patients coming from endemic countries [34]. A recent abstract from San Fransisco, California, noted that none of their Chagas tests came from the obstetrician/gynecology service [27]. It is important to identify mothers with Chagas disease and to identify risk factors for vertical transmission that are associated with increased vertical transmission, such as maternal parasitic load [12]. There is also recent evidence that there are parasite factors, such as haplotypes, that influence the risk of vertical transmission [41].

Neonates are at an increased risk of being neglected since they do not present the usual signs and cannot express their symptoms [36]. In our study, the youngest patient being evaluated was 4 years old; thus, no testing was performed in the neonatal period, similar to what was reported in San Francisco [27]. This is even more important given that if Chagas disease is found in infants the treatment is considered curative, with 90% cure rates in those treated within the first year of life [42]. A possible explanation is that there is a lack of awareness amongst neonatologists regarding this curable disease. To our knowledge, no survey of neonatologists has been conducted to date, but there is a need to understand how well neonatologists are aware of this neglected disease. Treating congenital Chagas has the potential to eliminate further morbidity of this disease in a person’s lifetime and could eliminate further generational transmission.

Despite the signs of Chagas disease’s presence in Louisiana, our results are the first to report the undertesting for Chagas disease from the healthcare system for both the adult and the pediatric population. It is important to determine the undertesting of this disease given that we know that informational sessions can improve the testing frequency of this disease [43].

Our study highlights the need to raise awareness of Chagas disease among health providers and the need to advocate for health promotion initiatives at all levels of the healthcare system in Louisiana. Future studies should help evaluate the reasons for the undertesting of the disease, evaluate different educational methods to increase awareness among providers, evaluate testing of congenital cases, and evaluate Chagas disease testing in multiple cities across the US.

## Figures and Tables

**Table 1:** Chagas Disease Testing in New Orleans.

Hospital	UMCNO	CHNOLA	Total
Number of tests ordered	33	6	39
Total patients	23	4	27
Male patients (%)	16 (70%)	3 (75%)	19 (70%)
Age of patients (median and range)	41 (20–75)	16.5 (4–17)	40 (4–75)
Hispanic patients (%)	18 (78%)	2 (50%) *	20 (74%) *
Presumed positive patients (%)	2 (8.7%)	0 (0%)	2 (7.4%) **

Notes: UMCNO: University Medical Center in New Orleans; CHNOLA: Children’s Hospital New Orleans. * One patient declined his ethnicity to be reported; ** both patients who were positive for Chagas disease were Hispanic, and the results were not confirmed.

## Data Availability

Available on reasonable request.
